# The immune and metabolic treatment approach of using testosterone on mice models of liver injury

**DOI:** 10.3389/fphar.2023.1219709

**Published:** 2023-08-08

**Authors:** Johnny Amer, Ahmad Salhab, Hadeel Snobar, Yazan Alhabil

**Affiliations:** ^1^ Department of Allied Sciences, Faculty of Medicine and Health Sciences, An-Najah National University, Nablus, Palestine; ^2^ Department of Biomedical Sciences, Faculty of Medicine and Health Sciences, An-Najah National University, Nablus, Palestine; ^3^ Department of Higher Education, Faculty of Medicine and Health Sciences, An-Najah National University, Nablus, Palestine; ^4^ Department of Medicine, Faculty of Medicine and Health Sciences, An-Najah National University, Nablus, Palestine

**Keywords:** liver injury, testosterone, NK cells, IL-6, IL-6 receptor

## Abstract

**Background:** Natural killer (NK) cells showed an anti-fibrotic effect; however, their function is thought to be impaired in advanced liver injury. In the current study, we aimed to assess the immune and metabolic impact of testosterone on mice models of liver injury.

**Methods:** Carbon-tetrachloride induced liver fibrosis male mice models was i.p injected for 2 weeks (acute) and 4 weeks (chronic) (*n* = 36). Testosterone (4 mg/kg mouse body weight) was injected i.p. following the first week of the acute model of CCl_4_ and following the second week of the chronic model of CCl_4_. At the end of the experiments, mice were sacrificed, and serum was collected for assessing liver enzymes of ALT and AST, as well as inflammatory markers of IL-6, metabolic makers of C-peptide levels, and lipid and glucose profiles. Livers were harvested and used for histological assessments for inflammation and fibrosis. Fibrosis profiles from liver extracts, αSMA and Collagen III, were assessed by RT-PCR. Moreover, liver tissue-resident NK cells were isolated and evaluated for their activity by assessing INF-γ and IL-6 receptors using ELISA and flow cytometry, respectively.

**Results:** Serum ALT, AST, and IL-6, as well as metabolic assessments of cholesterol, triglyceride, C-peptide, fasting blood sugar, and fibrotic profiles, were linearly correlated with disease progressions. Histological characterization of the liver was worsened in the chronic model of liver injury. Testosterone-treated mice exhibit a significant reduction in collagen depositions with less dense fibrosis tissue associated with reduced liver injury enzymes and metabolic markers in both the acute and chronic CCl_4_ mice models in favor of the latter one (*p* < 0.05). Moreover, testosterone treatments displayed a significant decrease in serum IL-6 of 2.4-fold (*p* = 0.0001) and 2.3-fold (*p* = 0.0003) in the acute and chronic models, respectively (*p* = 0.002), and data showed an increase in INF-γ release from NK associated with a reduction in their IL-6 receptor expressions (*p* < 0.05).

**Conclusion:** Our results indicated effects of testosterone on mediating a decreased expressions of NK IL-6 receptors and consequently inducing their activation; which in part, could explain the amelioration of liver injury. Our data suggest an anti-inflammatory and anti-fibrotic treatment approach of using testosterone for delaying disease progressions.

## Introduction

Testosterone has been shown to adjust carbohydrate, fat, and protein metabolisms and affect muscle growth and adipogenesis (Yassin et al., 2019). As the major male circulating androgen, testosterone provides a variety of biological processes in many tissues and organs such as the muscle and bones (Kelly and Jones, 2013). Testosterone therapy has become a moderately common treatment for men suffering from testosterone deficiency (Morgentaler and Traish, 2020). However, testosterone therapy is the standard practice in otherwise healthy hypogonadal men with prostate cancer history (Natale et al., 2021). Up to 90% of men with liver cirrhosis have decreased serum testosterone levels, which continue to decline as the liver condition worsens (Sinclair et al., 2015). Advanced liver illness shares many characteristics with hypogonadal males, such as sarcopenia, osteoporosis, gynecomastia, and reduced libido (Yurci et al., 2011). Al-Qudimat A *et al.* suggest that long-term testosterone therapy in hypogonadal men improves liver function (Al-Qudimat et al., 2021). However, it is not fully proven how much testosterone deprivation contributes to the symptoms of severe liver disease. Natural killer (NK) cells play critical roles in innate immune defense against bacterial, viral, and parasitic pathogens, as well as tumor suppression through natural cytotoxicity and cytokine secretion (Fasbender et al., 2016; Wei et al., 2022). Manipulation of NK cell activation has become a potential liver fibrosis immunotherapy, such as the adoptive transfer of allogeneic NK cells, genetic-engineered NK cells, and NK cell-targeted chemotherapy (Zhang et al., 2022). In our current study, we aimed to assess the molecular and metabolic aspects of testosterone and their modulatory effects on liver tissue-resident NK cells’ phenotype activations in mice models of liver injury.

## Materials and methods

### Experimental design

C57BL/6 male mice at 12 weeks of age and weighing 22.5 ± 1.5 g received care according to the An-Najah National University ethical guidelines. All animal protocols were approved by the institutional animal care ethical committee (Ref: Med. Oct/2018/59).

### Testosterone effects on liver injury

Liver injury mice models were induced using carbon tetrachloride (CCl_4_; Sigma, C-5331) introduced by i.p injections of 0.5 μL pure CCl_4_/g body weight (one to nine dilution in corn oil) twice a week for 2and 4 weeks as acute and advanced chronic liver injury. During the liver injury duration, 2 weeks in the chronic model and 1 week in the acute model, mice were i.p injected with testosterone (Merck; T1500; purity ≥98%) in the concentration of 100 µg/mouse [4 mg/kg mouse body weight] twice a week for the remaining weeks. In all experiments, mice were sacrificed 2 days after the final CCl_4_ injection. To this end, the animals were weighed and anesthetized with inhaled 5% isoflurane for 10 s before cervical dislocation.

### Mice groups

The following mice groups were included: 1) Naive mice (mice untreated with neither CCl_4_ nor testosterone), 2) mice group treated with testosterone only, 3) CCl_4_-treated mice—acute liver injury mode (2-week injections), 4) CCl_4_-treated mice—acute liver injury and treated with testosterone, 5) CCl_4_-treated mice—chronic liver injury mode (4-week injections), and 6) CCl_4_-treated mice—chronic liver injury and treated with testosterone. Each experimental group included six mice and the experiment was repeated three times (a total of 108 mice).

### Histological assessment

The posterior third of prostate and liver tissues were fixed in 3% formalin overnight and then embedded in paraffin in an automated tissue processor. Sections (7 μm) were stained with H&E to assess steatosis, area regions of necroinflammation, and apoptotic bodies, and with 0.1% Sirius red F3B in a saturated picric acid stain (Abcam, ab150681) to visualize connective tissue. A veterinary pathologist assessed all histopathological findings and reported assessments and the grade of the assessment.

### Serum biochemical assessments

Peripheral blood from the heart that was collected on the sacrifice day was centrifuged at 5,000 rpm for 15 min at 4°C to obtain the serum. Serum ALT (Abcam; ab285263), AST (Biocompare; MBS2019147), Fasting blood sugar (Biocompare; MBS7200879), C-peptide (Biocompare; MBS007738), cholesterol (Abcam; ab285242), and triglycerides (Biocompare; MBS726589) were determined using ELISA kits according to the manufacturer’s protocols.

### RNA isolation, cDNA preparation, and real-time PCR

RNA was obtained from liver tissue using trizol buffer (Bio-Lab; Cat# 90102331). Liver tissues were homogenized at RT, and 0.2 mL chloroform (Bio Lab; Cat# 03080521) was added. The samples were then incubated for 15 min at room temperature and centrifuged (1,400 rpm) for 15 min at 4°C. For RNA precipitation, the supernatant in each sample was transferred to a new micro-centrifuge tube, and 0.5 mL of isopropanol (Bio Lab; Cat# 16260521) was added, followed by 10 min incubation at 25°C. The tubes were then centrifuged (12,000 rpm) for 10 min at 4°C, the supernatants were removed, and 1 mL of 75% ethanol was added to the pellet, followed by centrifugation (7,500 rpm) for 5 min. The pellets were air-dried at room temperature for 15 min, 50 μL of DEPC was added, and the samples were heated for 10 min at 55°C. RNA purification from NK cells was assessed using RNeasy plus mini kit (CAT# 74034) according to the manufacturer’s guidelines. cDNA was obtained using a High-Capacity cDNA Isolation Kit (R&D; Cat# 1406197). RT-PCR reactions were performed using TaqMan Master Mix (Applied Biosystems; Cat# 4371130) to quantify *αSMA* and *collagen III* mRNA levels. Results were normalized to *gapdh* as a housekeeping gene and analyzed using QuantStudio™ 5 Real-Time PCR System.

### ELISA

Serum levels of testosterone and estradiol were assessed using abcam: ab285350 and Creative diagnostics: DEIA04927, respectively. Moreover, intracellular IL-6 and IFN-γ concentrations were assessed using Human IL-6 Quantikine ELISA Kit (R&D; D6050) and Human IFN-γ Quantikine ELISA Kit (R&D; 285-IF) according to the manufacturer’s protocols.

### Liver tissue-resident NK (trNK) cells isolation

The livers were extracted and placed in Petri dishes containing 10 mL of DMEM medium (Biological Industries; Cat# 01–055-1A). The liver tissue was thoroughly dispersed using a stainless-steel mesh, and the cells were collected along with the medium and transferred to 50 mL tubes containing 10 mL of DMEM. Subsequently, the cells were cautiously moved to new tubes containing Ficoll (Abcam; Cat# AB18115269) and subjected to centrifugation at 1,600 rpm for 20 min at 20°C. The resulting supernatant from each tube was transferred to fresh tubes and centrifuged again at 1,600 rpm for 10 min at 4°C. Following the second centrifugation, the cell pellet in each tube was resuspended in 1 mL of DMEM to isolate and purify NK cells using the Stem Cells kit (Cat# 19665).

### Flow cytometry

The trNK cells isolated from harvested mice livers were diluted to a concentration of 1 million cells per milliliter in a saline buffer supplemented with 1% bovine albumin (Biological Industries; Cat# 02–023-5A). Subsequently, the cells were labeled with the following antibodies. Anti-mouse NK1.1 (murine NK cell marker) (Biogems; Cat# 83712–70), anti-CD49a (MACS; Lot# 5150716246), anti CD49b (MACS; Lot# 5150716256), anti-mouse lysosomal-associated membrane protein-1 (CD107a; NK1.1 cells cytotoxicity marker, eBioscience, Cat# 48–1071), and anti-IL-6 R (R&D; Cat# 48–1044) were used. All antibodies were incubated for 40 min at 4°C. pHSCs (106 cells/mL) were stained with rabbit anti-mice αSMA (R&D; IC1420P). The cells were washed with 0.5 mL staining buffer and fixed with 20 mL 2% paraformaldehyde. All stained cells were analyzed with a flow cytometer (BD LSR Fortessa™, Becton Dickinson, Immunofluorimetry systems, Mountain View, CA).

### Statistical analysis

Statistical differences were analyzed with a two-tailed unpaired Student’s t-test (for comparisons between two groups) or one-way or two-way analysis of variance (ANOVA; one-way ANOVA with the Newman‒Keuls *post hoc* test for comparisons among multiple groups) with GraphPad Prism 9.0 (GraphPad Software, La Jolla, CA). A *t*-test of *p*-value ≤0.05 was considered statistically significant and was calculated as the difference in means between two variables. A Mann-Whitney *U* test was performed to evaluate whether the mice metabolic panel elements (ALT, AST, Cholesterol, Triglyceride, and FBS levels) were altered following testosterone treatment in both the acute and chronic liver injury groups. The correlation coefficient r test and normality test [the Shapiro-Wilk test] were used (*p*-value ≤ 0.05 is considered statistically significant). The experiment was repeated three times, with each repetition consisting of 10 sample replicates. Results are presented as mean ± SD or as average means of experimental replicates ±SD.

## Results

### Testosterone ameliorates inflammatory and fibrotic profiles of mice models of CCl_4_ liver injury 

**FIGURE 1:**
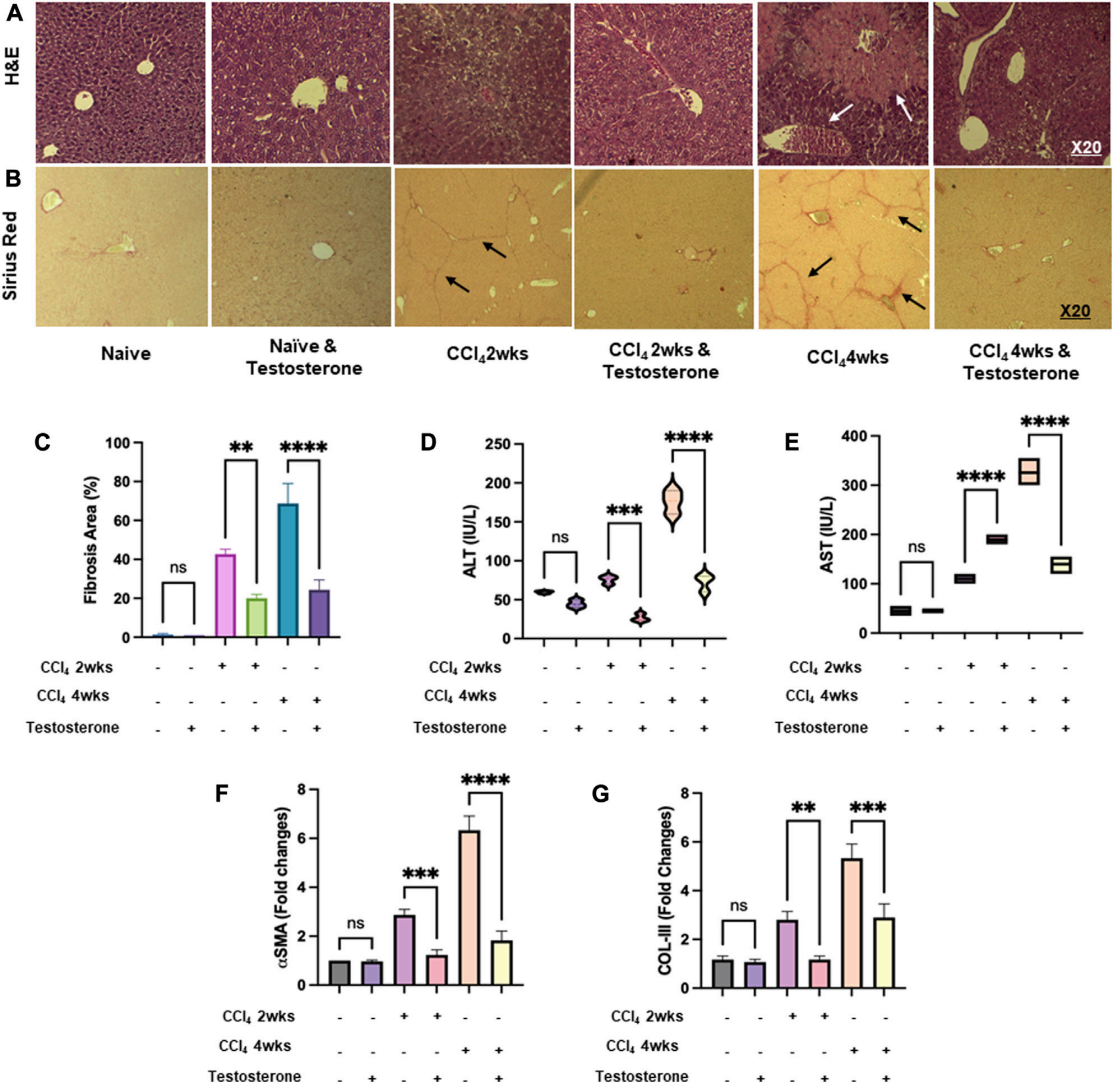
Testosterone alleviates histopathological and biochemical findings of liver fibrosis. Liver injury was induced in C57/BL male mice for 2 and 4 weeks and was compared with naive counterparts. Testosterone was administered via *i.p* injection for 1 week and 2 weeks, starting at weeks 1 and 2 of the acute and chronic CCl_4_ models, respectively, as described in Materials and Methods. Representative images of immunohistochemical liver staining sections of **(A)** H&E and **(B)** Sirius red, shown at an original magnification of ×10. The quantification of liver histology assessments is presented in **(C)** as the average ±SD for each group (six mice per group). Serum markers of liver injury **(D)** ALT and **(E)** AST were measured. mRNA markers of liver fibrosis of **(F)** αSMA and **(G)** collagen III were assessed. Each experiment was repeated three times to ensure reliability and reproducibility. [**p = 0.01, ***p = 0.005, and ****p = 0.0001]. A Mann-Whitney *U* test was performed to evaluate whether the liver injury mice marker (ALT and AST) was altered by testosterone treatment in both the acute and chronic CCl_4_-injected groups. The results demonstrated significant values in both groups. Therefore, the null hypothesis is rejected. Data is normally distributed (alpha = 0.05).

Liver sections from mice with acute and chronic CCl_4_-induced liver injury were evaluated for liver injury and phenotypic changes after treatment with testosterone. Representative images of H&E staining (Figure 1A) and Sirius Red staining (Figure 1B) of liver sections depicting acute and chronic liver injury are shown. The H&E staining of CCl_4_-treated livers revealed centrilobular hepatocytes that were swollen, along with extensive necrotic areas containing a high number of infiltrating inflammatory cells (white arrows left) and steatosis (white arrows right), indicating the presence of a chronic CCl_4_ model. However, in mice treated with testosterone, there was a delay in the appearance of these histological findings, and a significant reduction in both microvascular and macrovascular steatosis was observed specifically in the chronic model. Sirius red staining of livers from the CCl_4_ mice exhibited increased collagen deposition in perisinusoidal areas in both the acute and chronic CCl_4_ models (black arrows), but the impact of collagen depositions was more pronounced in the chronic model. Treatment with testosterone resulted in a remarkable reduction in the dense fibrous tissue of the stained area compared to the vehicle-treated mice. Figure 1C summarizes a detailed histology scoring system for H&E and fibrosis assessments (Scheuer, 1991; Ishak et al., 1995; Bedossa and Poynard, 1996; Wallace et al., 2015). Biochemical markers were also assessed in our mice groups. Serum inflammatory profiles of ALT (Figure 1D) and AST (Figure 1E) were elevated in both acute and chronic CCl_4_ models in favor of the latter one. A significant amelioration in ALT levels of 2.1-folds and 3.6-folds were achieved in the acute and chronic CCl4 model, respectively, following testosterone treatment (*p* < 0.05). Similar effects of testosterone treatment were achieved only in the chronic CCl4 model of 2.6-folds while no effects were seen in the acute model. To confirm liver fibrosis in CCl_4_-induced mice, fibrosis markers were quantified by assessing liver αSMA (Figure 1F) and collagen III (Col III) (Figure 1G) using RT-PCR. The data showed a significant increase in αSMA and Col III levels in both the acute and chronic CCl_4_ models compared to the vehicle group, with a 1.2-fold increase in the acute model and a 4.2-fold increase in the chronic model for αSMA, and a 1.2-fold increase in the acute model and a 3.2-fold increase in the chronic model for Col III (*p* = 0.002). However, mice with liver fibrosis receiving testosterone treatment exhibited significant reductions in αSMA and Col III levels, with a 1.2-fold decrease and a 1.3-fold decrease, respectively, (*p* < 0.03) in the acute CCl_4_ model, and a 2.2-fold decrease and a 2.3-fold decrease (*p* < 0.03) in the chronic CCl_4_ model. The results obtained from both RT-PCR and histology assessments were comparable and clearly indicated an improvement in liver injury, inflammation, and fibrosis in liver sections following treatment with testosterone. To elucidate the effects of testosterone on alleviating histopathological findings of liver sections, we assessed serum testosterone and estradiol levels. Testosterone could be metabolized to estradiol through aromatization (Roncati et al., 2016). In male models, testosterone is the major source of plasma estradiol, the main biologically active estrogen, 20% of which is secreted by the testes. Plasma estrone, 5% of which is converted to plasma estradiol, originates from tissue aromatization, mainly adrenal and androstenedione (Vermeulen et al., 2002).

Supplementary Figure S1 displays serum testosterone (A) and estradiol (B) following testosterone treatment. Low serum testosterone levels were significantly obtained following CCl_4_ inductions compared to untreated mice and were positively correlated with the severity of liver fibrosis. Testosterone treatment elevated serum testosterone levels and was comparable in all mice groups including the control group (untreated mice). In parallel, the same pattern of estradiol serum levels was achieved in untreated mice and showed reductions in their levels along severities of liver fibrosis. Testosterone treatment induced elevated estradiol levels and was positively correlated to liver fibrosis severity of chronic CCl_4_ inductions (2.3-folds, *p* = 0.0001). Although estradiol levels increased following testosterone treatment, they remained within the normal range (Ström et al., 2012), highlighting the importance of testosterone in delaying liver fibrosis.

### Testosterone improves metabolic assessments of mice models of liver injury

**FIGURE 2:**
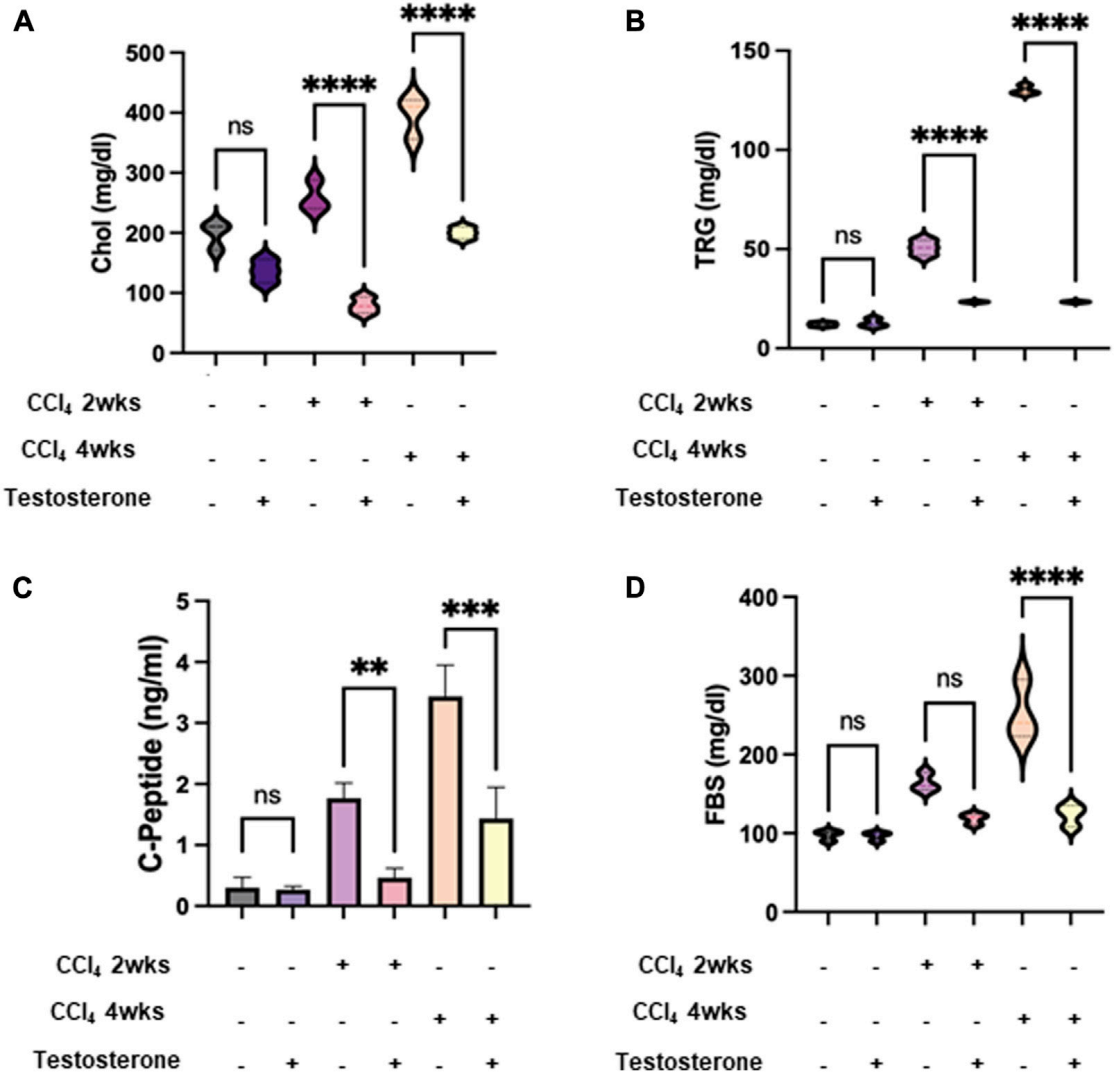
Testosterone improved the perturbed metabolic profile in CCl_4_-induced animals. Metabolic markers of lipid and glucose profile of serum levels of **(A)** cholesterol; CHOL, **(B)** triglyceride; TRG, **(C)** C-peptide, and **(D)** fasting blood sugar; FBS were assessed following 16 h of fasting. Each measurement was repeated three times and data were represented as mean ± SD [*p = 0.01, ***p = 0.005, and ****p = 0.0001]. A Mann-Whitney *U* test was performed to evaluate whether the metabolic panel elements (Cholesterol, Triglyceride, C-peptide, and FBS levels) were altered by testosterone treatment in both the acute and chronic CCl_4_-injected groups. The results demonstrated significant values in both groups. Therefore, the null hypothesis is rejected. Data is normally distributed (alpha = 0.05).

CCl_4_ administration in C57BL/6J mice exacerbates high cholesterol levels and induces steatohepatitis changes in the liver. Previous research by Bassi et al. (2005) demonstrated that CCl_4_ leads to increased hepatic lipid profiles of cholesterol, fatty acids, and triglycerides in both chronic and acute treatments in rats. Based on these findings, we utilized this model to examine the metabolic outcome markers of lipid and glucose profiles following treatment with testosterone. Our mice models exhibited perturbations in the metabolic profile of CCl_4_-induced animals. In Figure 2, we observe elevated serum levels of cholesterol (Figure 2A), triglycerides (Figure 2B), C-peptide (Figure 2C), and fasting blood sugar (FBS) (Figure 2D) in both the acute and chronic treatments of CCl_4_ mice, with a more significant impact in the chronic model. However, the liver injury mice treated with testosterone demonstrated lower serum levels of cholesterol, triglycerides, and C-peptide compared to the control groups receiving the vehicle, while also displaying a reduction in FBS levels (Figure 2D).

### Testosterone decreases IL-6 concentrations in mice models of CCl_4_ liver injury

**FIGURE 3:**
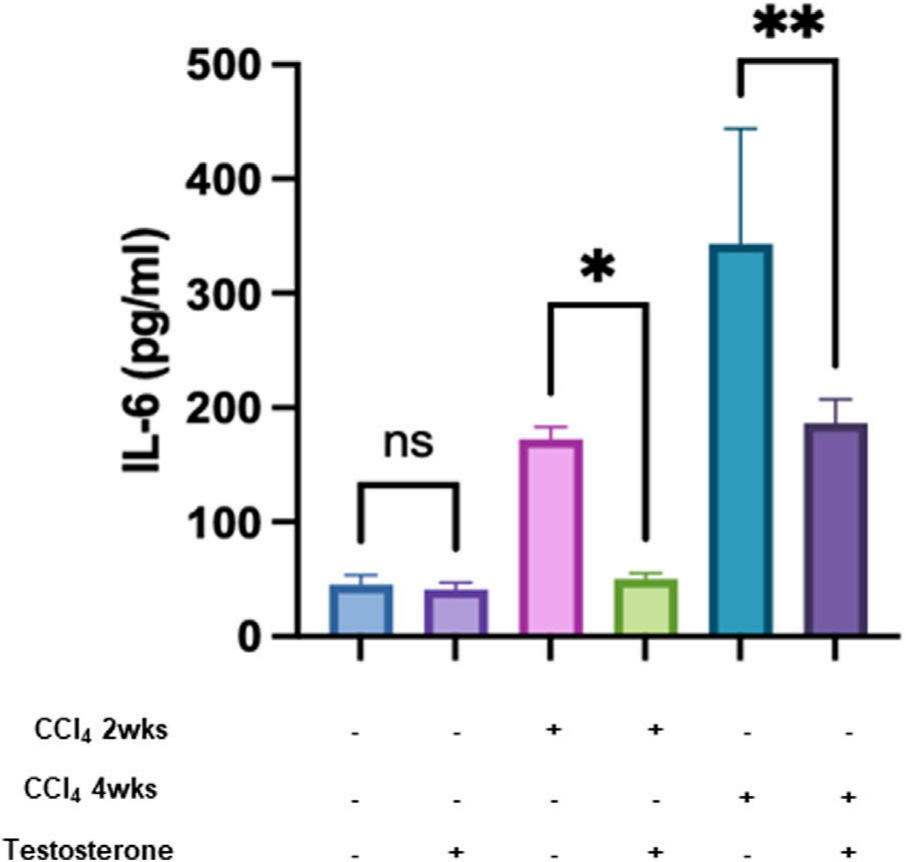
Testosterone displays an inflammatory effect by reducing inflammatory cytokine. Pro-inflammatory cytokine levels of IL-6 were measured in all groups in triplicates. Data were analyzed using a Quantibody Q-Analyzer and an Excel-based program; results are presented in pg/mL. Data show mean ± SD. [*p = 0.04, **p = 0.012].

Altogether, the above data indicate that testosterone has an anti-fibrotic effect, most probably due to its effects in ameliorating lipid and glucose profiles, both of which are risk factors contributing to fibrogenesis. Thus, our results indicate testosterone is a potential target for delaying and inhibiting liver injury by improving insulin sensitivity. To further explore the mechanism behind the antifibrotic effects of testosterone, we further assessed the inflammatory and immune contribution in alleviating liver injury. We assessed serum IL-6 levels, the activity of isolated liver tissue-resident NK (trNK) cells, and the expression of IL-6 receptors on trNK cells. Testosterone exhibited immune-modulating properties, supported by *in vitro* evidence suggesting its potential to suppress the expression of proinflammatory cytokines such as TNFα, IL-1β, and IL-6, while enhancing the expression of the anti-inflammatory cytokine IL-10 (Arslan et al., 2016). Furthermore, testosterone displayed anti-inflammatory effects by significantly inhibiting adipose tissue formation and downregulating the expression of various adipocytokines, including leptin, TNF-α, IL-6, and IL-1, while positively correlating with adiponectin levels. Conversely, low testosterone levels were associated with increased expressions of inflammatory markers. Data presented in Figure 3 shows that both naive mice treated and untreated with testosterone had comparable low levels of serum IL-6 of 65 ± 10 pg/mL (*p* = ns). Serum IL6 showed increased levels within liver injury severities of 180 ± 24 pg/mL and 345 ± 52 pg/mL in the acute and chronic models, respectively (*p* = 0.002). Testosterone treatments exhibited a significant decrease of 2.4-fold (*p* = 0.0001) and 2.3-fold (*p* = 0.0003) in the acute and chronic models, respectively (*p* = 0.002). Testosterone has an anti-inflammatory effect due to the reduction of inflammatory cytokines (Bianchi, 2019).

### Testosterone-treated CCl_4_-mice showed liver recruitment of trNK cells and restored their activity

**FIGURE 4:**
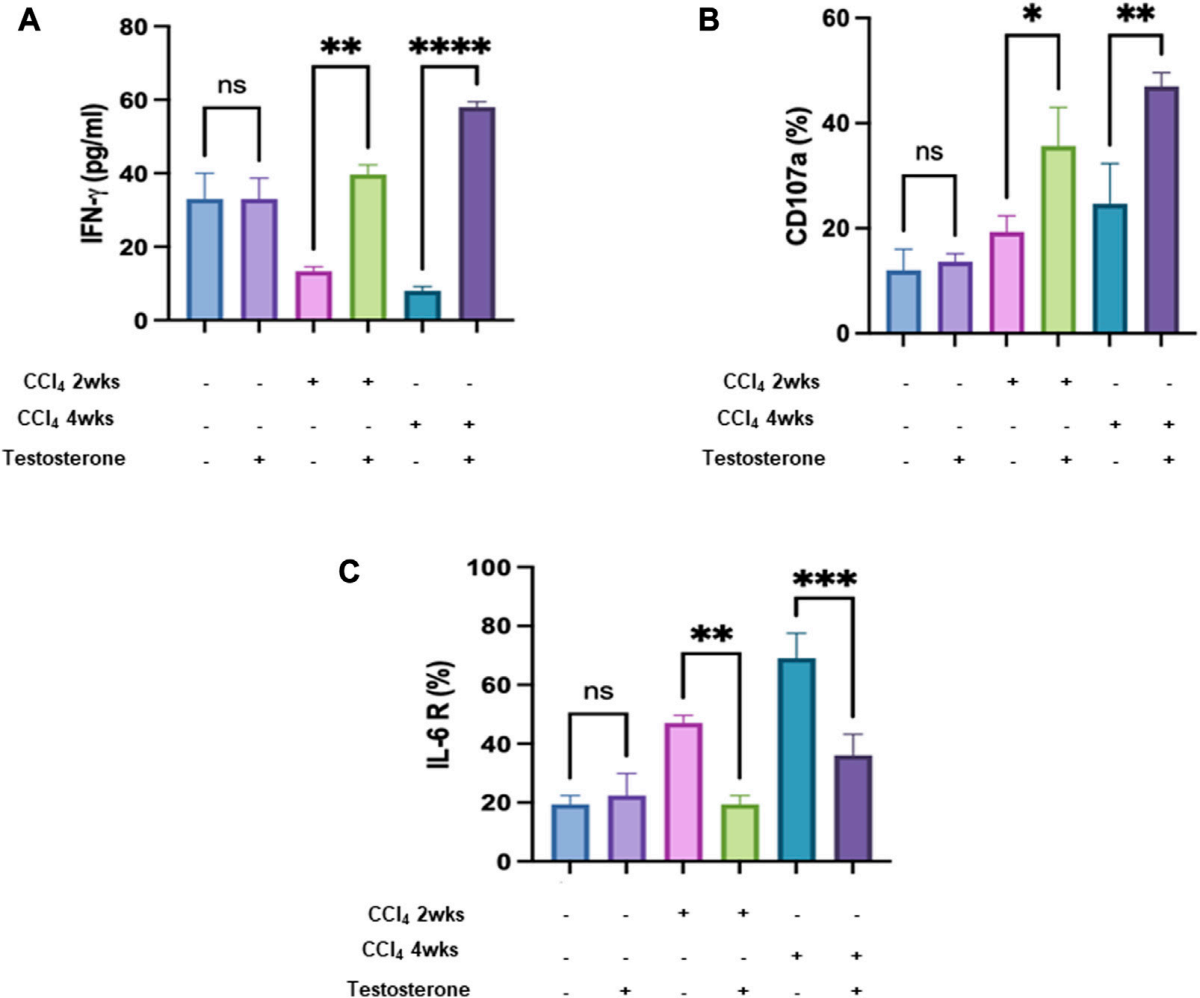
Testosterone ameliorates liver injury by reducing NK IFN γ and improving liver trNK activity. **(A)** ELISA showed secreted IFN-γ in mice in all mice groups. Flow cytometry analysis data demonstrated trNK **(B)** CD107a and **(C)** IL-6R percentages. (Each experiment was repeated three times and data was represented as mean ± SD. [*p = 0.05, **p = 0.01, ***p = 0.005, and ****p = 0.0001].

The immune system plays a crucial role in the process of tissue healing. Consequently, managing the immune system is essential for effective planning of the healing process. In our study, we examined liver tissue-resident NK cells (trNK) isolated from the different mice groups. NK cells have been shown to possess antifibrotic properties by eliminating activated hepatic stellate cells (HSCs) (Muhanna et al., 2008). However, it is believed that their functionality may be impaired in cases of advanced liver injury (Amer et al., 2018; Salhab et al., 2020). Figure 4A shows an inverse correlation between trNK secretions of IFN-γ and liver injury severity of a 2-fold decrease (*p* = 0.003). Following testosterone treatment, trNK secretions of IFN-γ showed higher levels of 2.1 and 6.3 folds in the acute and chronic models, respectively. Regarding the correlation coefficient r test, Figure 4A’s Pearson’s correlation coefficient (−0.675) provides evidence for a large inverse strength of association between the variables.

The same data patterns were obtained using NK activation markers of CD107a (Figure 4B). Moreover, to further associate trNK activatory effects following testosterone treatment with IL-6 receptor, flow cytometry analysis was performed as indicated in Materials and Methods. Figure 4C shows a significant reduction of 1.6-fold and 2.5-fold in IL-6 receptor in the acute and chronic liver injury model, respectively, compared to mice receiving the vehicle (*p* = 0.0001).

Our results undoubtedly indicate the impact of testosterone as a potential therapy as they exhibit antifibrotic and anti-inflammatory effects by reducing αSMA mediated by increased NK activity and reductions in IL-6 receptor and could be of beneficial influence for patients with advanced liver injury.

## Discussion

In this study, we investigated the potential beneficial effect of testosterone on hepatic histopathological, immunological, and biochemical changes in a CCl_4_-induced mice model. Several studies have shown a relationship between testosterone and liver injury (Al-Qudimat et al., 2021). Even though testosterone treatment improves liver injury and metabolic syndrome, and its effect on type two diabetes mellitus is well-documented (Sun et al., 2021), testosterone effects on hepatic injury are still limited. In the present study, we conducted an experimental prospective study on the effect of testosterone treatment on liver injury by assessing liver enzymes, metabolic and oxidative stress, and real-time PCR for the liver and prostate. A previous study used a CCl_4_ model to induce hepatotoxicity and showed precautionary and therapeutic management of these substances (El Naggar et al., 2015). In addition, previous studies showed that testosterone has an anti-inflammatory effect and improves liver injury (Ochayon and Waggoner, 2021).

Lipids and glucose are major risks in the pathogenesis of liver injury and are associated with morbidity due to diabetes and atherosclerosis. Free cholesterol activates HSCs, and the addition of cholesterol to a high-fat or methionine/choline-deficient diet leads to the accumulation of free cholesterol in HSCs, which accelerates experimental liver fibrosis (Tomita et al., 2014). High hepatocyte lipid droplet accumulation in the liver propagates liver injury and causes a storm of pro-inflammatory cytokines that can lead to steatosis and hepatocyte injury (Chin et al., 2020). Previous studies have demonstrated that testosterone has effects on various enzymatic pathways involved in fatty acid metabolism, glucose control, and energy utilization. These effects can be tissue-specific, with different outcomes observed in different fat depots, muscles, and liver. Testosterone treatment has been shown to have beneficial effects on obesity-related measures, which is partially attributed to its direct metabolic actions on adipose tissue and muscles, as well as potentially increasing motivation and energy levels, leading to more active lifestyles in obese individuals (Kelly and Jones, 2015).

CCl_4_ is well-known for its hepatotoxic effects (1). CCl_4_ disrupts the structural integrity of hepatocyte membranes and causes cellular death. This damage triggers inflammation and activates signaling pathways involved in tissue repair (Liu et al., 2021). Moreover, CCl_4_ generates reactive oxygen species (ROS) in the liver, leading to oxidative stress causing damage to cellular components, including lipids, proteins, and DNA. In addition, it activates immune cells, such as Kupffer cells, and infiltrates neutrophils, which release pro-inflammatory cytokines and chemokines (Khan et al., 2017). This inflammatory response contributes to tissue damage and can exacerbate liver injury (5). Prolonged or repeated exposure to CCl_4_ can lead to liver fibrosis, a condition characterized by excessive accumulation of scar tissue in the liver. CCl_4_ promotes the activation of hepatic stellate cells, which are responsible for producing excessive extracellular matrix components, leading to fibrosis development (Wang et al., 2021). Since liver diseases are typically multifactorial and involve a combination of genetic, environmental, and lifestyle factors, one of the limitations of the CCl_4_ mice model is the inability to fully capture the complexity of human liver diseases.

In our study, we observed improvements in metabolic markers such as cholesterol, triglycerides, C-peptide, and fasting blood sugar levels following testosterone treatment. These findings suggest that testosterone may contribute to improved liver histology and potentially slow down the progression of liver fibrosis by targeting the metabolic profile. Lipid and glucose dysregulation are major risk factors in the development of liver injury. Hepatic lipid accumulation can lead to systemic metabolic dysfunction by upregulating the expression of gluconeogenic peroxisome proliferator-activated receptor (PPAR) ligands, resulting in hyperglycemia, ketosis, and hyperlipidemia (Geng et al., 2021). Hepatic insulin resistance, characterized by impaired insulin-mediated suppression of glucose output from the liver, contributes to increased blood glucose levels (Jiang et al., 2020). Our data support the notion that testosterone administration leads to lower levels of cholesterol, LDL, triglycerides, and glucose compared to the non-treated group, indicating its potential role in regulating these metabolic parameters.

The present study revealed that there is a statistically significant relationship between testosterone hormone and liver injury by assessing injury markers of serum AST and ALT as indicators of hepatocellular injury. Several studies have demonstrated that high ALT and AST levels are correlated with a higher risk of liver fibrosis. High liver enzymes frequently signify liver cell inflammation or damage. Liver cells that are inflamed or wounded leak more substances into the bloodstream than usual, including liver enzymes, causing liver enzyme levels in the blood to rise (Corrales et al., 2006). In our present study, a significant decrease in ALT and AST was observed when administering testosterone exceeding the level at the baseline of the untreated group.

Our data indicate improved liver histology after testosterone treatment; thus, lowering the progression of liver injury could be partly achieved by targeting the metabolic profile. In our study, we evaluated the testosterone effect of selected real-time PCR results presenting collagen and alpha-smooth muscle in mice models that have liver injuries (acute and chronic injuries). Moreover, our data showed a decrease in collagen and αSMA levels following testosterone treatment. Previous research has shown that the severity of liver fibrosis in humans is associated with increased levels of αSMA and collagen. During the progression of fibrosis, the extracellular matrix (ECM) composition changes, and activated hepatic stellate cells (HSCs) play a role in inhibiting ECM degradation by secreting higher amounts of αSMA and collagen. The levels of αSMA and collagen were found to be significantly higher in cases of chronic fibrosis compared to acute or absent fibrosis (Munsterman et al., 2018), which is consistent with our generated data.

Our study provided compelling evidence for the amelioration of liver injury and the improvement of liver histology in terms of inflammation and fibrosis following testosterone treatment. These beneficial effects were accompanied by a decrease in the expression of IL-6 receptors on liver-resident NK cells and an increase in NK cell activity. These results suggest that the anti-inflammatory and anti-fibrotic effects of testosterone may be mediated, at least in part, through its impact on NK cells. Targeting the immune system, particularly NK cells, may be a promising strategy for delaying liver injury.

## Conclusion

Our study findings provide evidence for the therapeutic potential of testosterone in reducing liver injury and improving the histology of inflammation and fibrosis in the liver. This improvement was attributed to a decrease in NK IL-6 receptors, resulting in increased NK cell activity. These results highlight the immune-modulatory effects of testosterone, which are associated with its anti-inflammatory and anti-fibrotic properties. This suggests that testosterone may serve as a valuable approach in the treatment of liver conditions characterized by inflammation and fibrosis. It would be interesting to explore the practical implications of testosterone to fully assess its efficacy and safety for future clinical trials in humans.

## Data Availability

The raw data supporting the conclusion of this article will be made available by the authors, without undue reservation.

## Supplementary material

SUPPLEMENTARY FIGURE S1 Testosterone treatment elevated serum testosterone and estradiol levels. Serum **(A)** testosterone and **(B)** estradiol following testosterone treatment are shown using ELISA. Each experiment was repeated three times and data was represented as mean ± SD. [**p* = 0.05, ***p* = 0.01, ****p* = 0.005, and *****p* = 0.0001].
