# Supplementary figures and images for: The immune and metabolic treatment approach of using testosterone on mice models of liver injury
Source: Front Pharmacol. 2023 Aug 8;14:1219709. doi: 10.3389/fphar.2023.1219709 (PMC10442657; doi:10.3389/fphar.2023.1219709)

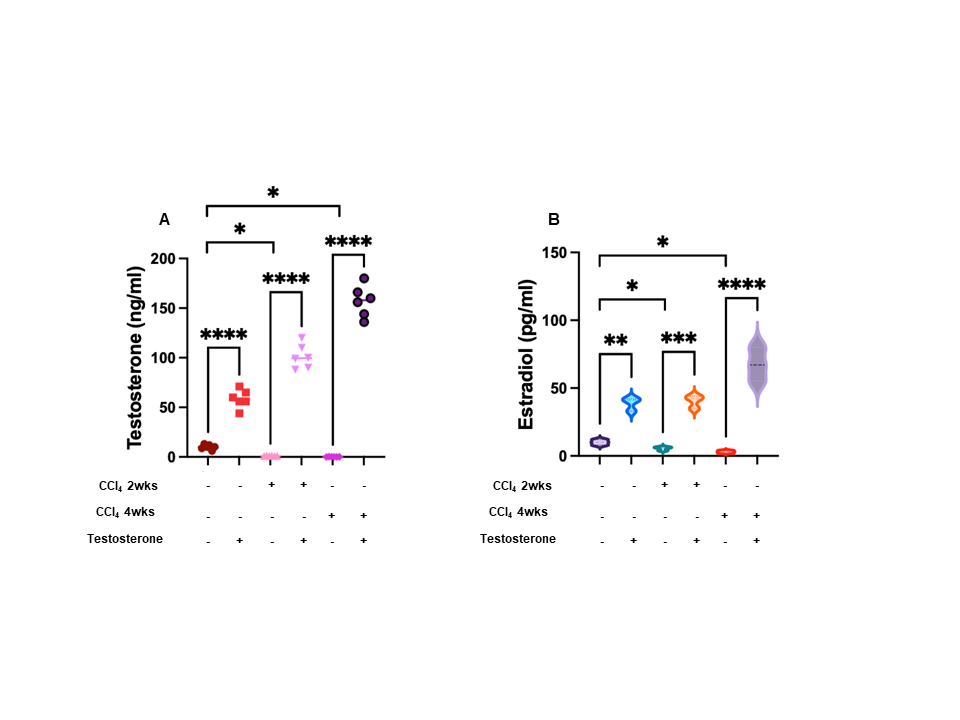

Supplement: Supplementary file 1 [file Image1.TIF]
